# Activin A Reduces GIRK Current to Excite Dentate Gyrus Granule Cells

**DOI:** 10.3389/fncel.2022.920388

**Published:** 2022-05-27

**Authors:** Fang Zheng, Maria Jesus Valero-Aracama, Natascha Schaefer, Christian Alzheimer

**Affiliations:** ^1^Institute of Physiology and Pathophysiology, Friedrich-Alexander-Universität, Erlangen-Nürnberg, Erlangen, Germany; ^2^Institute for Clinical Neurobiology, Julius-Maximilians-Universität Würzburg, Würzburg, Germany

**Keywords:** activin, hippocampus, GIRK current, action potential, dentate gyrus granule cells

## Abstract

Activin A, a member of the TGF-β family, is recognized as a multifunctional protein in the adult brain with a particular impact on neuronal circuits associated with cognitive and affective functions. Activin receptor signaling in mouse hippocampus is strongly enhanced by the exploration of an enriched environment (EE), a behavioral paradigm known to improve performance in learning and memory tasks and to ameliorate depression-like behaviors. To interrogate the relationship between EE, activin signaling, and cellular excitability in the hippocampus, we performed *ex vivo* whole-cell recordings from dentate gyrus (DG) granule cells (GCs) of wild type mice and transgenic mice expressing a dominant-negative mutant of activin receptor IB (dnActRIB), which disrupts activin signaling in a forebrain-specific fashion. We found that, after overnight EE housing, GC excitability was strongly enhanced in an activin-dependent fashion. Moreover, the effect of EE on GC firing was mimicked by pre-treatment of hippocampal slices from control mice with recombinant activin A for several hours. The excitatory effect of activin A was preserved when canonical SMAD-dependent signaling was pharmacologically suppressed but was blocked by inhibitors of ERK-MAPK and PKA signaling. The involvement of a non-genomic signaling cascade was supported by the fact that the excitatory effect of activin A was already achieved within minutes of application. With respect to the ionic mechanism underlying the increase in intrinsic excitability, voltage-clamp recordings revealed that activin A induced an apparent inward current, which resulted from the suppression of a standing G protein-gated inwardly rectifying K^+^ (GIRK) current. The link between EE, enhanced activin signaling, and inhibition of GIRK current was strengthened by the following findings: (i) The specific GIRK channel blocker tertiapin Q (TQ) occluded the characteristic electrophysiological effects of activin A in both current- and voltage-clamp recordings. (ii) The outward current evoked by the GIRK channel activator adenosine was significantly reduced by preceding EE exploration as well as by recombinant activin A in control slices. In conclusion, our study identifies GIRK current suppression *via* non-canonical activin signaling as a mechanism that might at least in part contribute to the beneficial effects of EE on cognitive performance and affective behavior.

## Introduction

Among the activin members of the transforming growth factor β (TGF-β) family, activin A, a homodimer of two βA subunits, is the most abundant and best characterized variant in the brain. Activin A was originally identified as a neurotrophic and neuroprotective factor, but more recent evidence from our and other laboratories implicated activin signaling also in the modulation of the neurophysiological underpinnings of cognitive functions, affective behavior, and drug addiction (Ageta and Tsuchida, 2011; Krieglstein et al., 2011; Hasegawa et al., 2014; Zheng et al., 2016; Link et al., 2016a; Bloise et al., 2019; Werner et al., 2020; Mitra et al., 2022). In general, the biological effects of activin A are mediated through heterotetrameric receptor complexes consisting of two activin-binding type II receptors (ActRIIA or ActRIIB) and two downstream-signaling type I receptors (mainly ActRIB). The latter act through the SMAD2/3 pathway to regulate the transcription of activin target genes (Bloise et al., 2019). In addition to this canonical pathway, activin receptors might also recruit other signaling systems, including mitogen-activated protein kinase (MAPK) and PKA/PKC, to exert non-genomic actions (Derynck and Zhang, 2003; Moustakas and Heldin, 2005; Tsuchida et al., 2009).

In the adult rodent brain, the basal level of activin A is low, but the factor is strongly responsive to both physiological and pathological stimuli. For example, high-frequency electrical stimulation of the perforant pathway to elicit long-term potentiation in the dentate gyrus (DG) of anesthetized rats induced a concomitant increase in activin βA mRNA in the potentiated region (Andreasson and Worley, 1995; Inokuchi et al., 1996). On the pathological side, epileptic activity, excitotoxic injury, and stroke all engendered even more pronounced rises in activin levels, which developed a strong neuroprotective potential (Tretter et al., 2000; Mukerji et al., 2009; Buchthal et al., 2018). In addition, activin A has been implicated in the regulation of mood disorders and advanced as an endogenous antidepressant. In support of this notion, activin signaling is targeted by antidepressant drugs and electroconvulsive therapy (ECT), the two prevailing treatment strategies for major depression (Dow et al., 2005; Ganea et al., 2012; Link et al., 2016b; Gergues et al., 2021). In a rodent model of ECT, the shock induced a massive up-regulation of activin A that is confined to the DG (Dow et al., 2005; Link et al., 2016b). Moreover, direct infusion of activin A into the DG, but not into the CA1 region, exerted antidepressant-like effects in the forced swimming test (Dow et al., 2005). Interestingly, exposure to a novel and enriched environment (EE) not only improves the performance in learning and memory tasks but also ameliorates depression-like behavior (Nithianantharajah and Hannan, 2006; Pang and Hannan, 2013; Mahati et al., 2016; Ohline and Abraham, 2019). In previous work, we found that brief sessions of EE exploration (2–12 h) induced a marked increase in the expression of activin βA mRNA in the mouse hippocampus (Link et al., 2016b). In contrast to the expression pattern after ECT, which was conspicuously restricted to DG, enhanced βA mRNA signals after EE were detected in the principal cell layers of all hippocampal regions, with some decline in intensity along the DG-CA3-CA1 circuit (Link et al., 2016b). In a recent study, we investigated the effects of elevated activin A levels after overnight EE on neural activity in mouse hippocampal CA1 region (Dahlmanns et al., 2022). Our main conclusion was that enhanced activin signaling serves to translate the behavioral stimulation afforded by EE into a range of complementary changes comprising cellular excitability, synaptic plasticity, and network activity (Dahlmanns et al., 2022). Here, we demonstrate that activin-dependent enhancement of cell firing after EE housing holds for DG granule cells, and we identified the underlying ionic mechanism and characterized the activin signaling pathway involved.

## Materials and Methods

### Animals

Male and female adult (2–4 months old) wild type (wt) C57BL/6J mice and transgenic mice expressing a dominant-negative mutant of activin receptor IB (dnActRIB) under the control of the CaMKIIα promoter (Müller et al., 2006) were used for experiments. Animals were housed under standard conditions with 12 h/12 h light/dark cycle and free access to water and food. All procedures were conducted in accordance with the Animal Protection Law of Germany and the European Communities Council Directive of November 1986 /86/609/EEC), and with the approval of the local government of Lower Franconia.

### Enriched Environment (EE)

As described previously (Link et al., 2016b), EE experiments were performed with pairs of mice, placed in large cages (60 × 38 × 18 cm) equipped with shelters, toys, and tunnels. EE housing lasted overnight from 9 p.m. to 9 a.m.

### Electrophysiological Recordings From Brain Slices

Transverse dorsal hippocampal slices (350 μm thick) were prepared from mice anesthetized with sevoflurane or isoflurane. Brain slices were cut in ice-cold sucrose-based artificial cerebrospinal fluid (aCSF) containing (in mM) 75 sucrose, 87 NaCl, 3 KCl, 0.5 CaCl_2_, 7 MgCl_2_, 1.25 NaH_2_PO_4_, 25 NaHCO_3_, and 10 D-glucose, and were then incubated in same solution for 10 min at 35°C. Slices were kept in aCSF containing (in mM) 125 NaCl, 3 KCl, 1 CaCl_2_, 3 MgCl_2_, 1.25 NaH_2_PO_4_, 25 NaHCO_3_, and 10 D-glucose at room temperature. Individual slices for recording were transferred to a submerged chamber perfused with normal aCSF with 1.5 mM MgCl_2_ and 2.5 mM CaCl_2_ at 31 ± 1°C. All solutions were constantly gassed with 95% O_2_–5% CO_2_. Whole-cell recordings of visualized DG granule cells (GCs) in the suprapyramidal blade were performed in cells located in the outer part of the granule cell layer (i.e., close to the molecular layer). Patch pipettes were filled with (in mM) 135 K-gluconate, 5 HEPES, 3 MgCl_2_, 5 EGTA, 2 Na_2_ATP, 0.3 Na_3_GTP, 4 NaCl (pH 7.3). All potentials were corrected for liquid junction potential. Signals were filtered at 6 kHz (for current clamp) or 2 kHz (for voltage clamp) and sampled at 20 kHz using a Multiclamp 700B amplifier in conjunction with Digidata 1440A interface and pClamp10 software (Molecular Devices, Sunnyvale, CA).

In current-clamp mode, DG granule cells normally do not discharge at resting membrane potential (RMP). To test neuronal excitability, square depolarizing gpulses at different current intensities (50–200 pA, for 1 s) were used to elicit action potentials (AP) at rest or at −70 mV by injecting current. The membrane input resistance (R_N_) was determined with 20 pA hyperpolarizing pulse (1 s). To avoid sampling immature granule cells, only cells with R_N_ less than 450 MΩ were included. To examine the acute effect of recombinant activin A (50 ng/ml; R&D System) on granule cell excitability, APs were elicited by a depolarizing ramp protocol (2 s) with individually adjusted ramp current, with membrane potential set to −70 mV before drug application. In voltage-clamp mode, a voltage ramp ranging from −50 to −140 mV at a rate of 0.1 mV/ms was used to evaluate G protein-gated inwardly rectifying K^+^ (GIRK) channel activity, mostly in the presence of tetrodotoxin (TTX ; 1 μM) to block synaptic activity. Agonists for GABA_B_ receptors and adenosine receptors were used to activate GIRK channels in DG granule cells. The evoked GIRK current was obtained by subtracting the control current response from that during the drug’s peak effect. Tonic GIRK current was determined as the difference in the steady-state current before and during the application of the specific GIRK channel blocker tertiapin Q (TQ, 200 nM). Unless otherwise stated, drugs and chemicals were obtained from Tocris Bioscience (Bio-techne GmbH, Wiesbaden, Germany) and Sigma-Aldrich Chemie GmbH (Steinheim, Germany).

### Enzyme-Linked Immunosorbent Assay (ELISA)

Mice with and without overnight EE housing were anesthetized with sevoflurane or isoflurane and the brains were rapidly dissected. The isolated hippocampi were mechanically broken down with a magnetic homogenizing ball for 30 s in lysis buffer containing 0.32 M sucrose, 5 mM Tris-HCl (pH 8.0), and protease inhibitor cocktail (Sigma Aldrich), and homogenates were centrifuged at 13,000× *g* at 4°C for 10 min (twice). Supernatants were collected and activin A levels were assayed by ELISA kit (R&D Systems; Bio-techne GmbH, Wiesbaden-Nordenstadt, Germany) according to the manufacturer’s instructions.

### Statistical Analysis

Data analysis was performed offline with Clampfit 10.6 (Molecular Devices, CA, USA). Data were expressed as means ± SEM. Statistical comparisons of data were performed with OriginPro 2018G (OriginLab Corporation, MA, USA), using ANOVA or Student’s *t*-test as appropriate. Significance was assumed for *p* < 0.05.

## Results

### EE-Induced Increase in Granule Cell Excitability Is Linked to Activin Signaling

**Figure 1:**
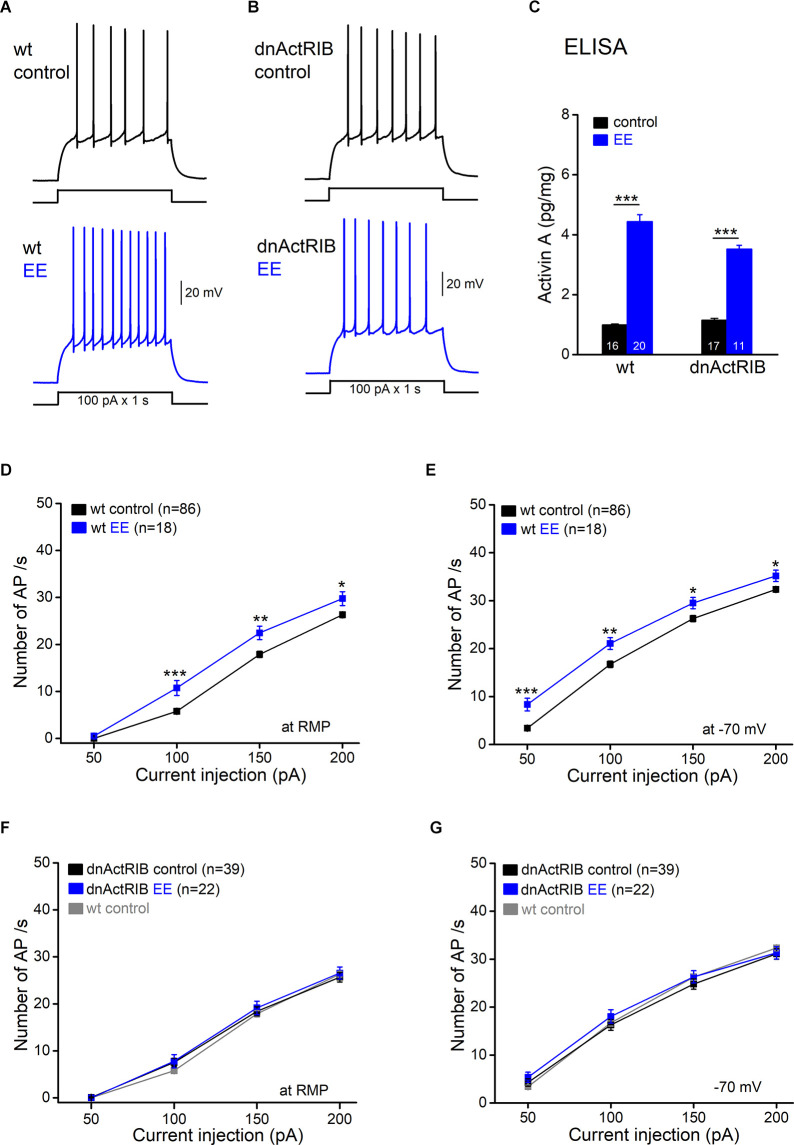
Excitatoryeffect of enriched environment (EE) on dentate gyrus granule cellfiring is blocked in dnActRIB mice. **(A,B)** Whole-cellcurrent-clamp recordings from granule cells from wild-type (wt; **A**) and dnActRIB **(B)** mice with and without overnight (12 h) EE housing illustrate the repetitive action potentials (APs), evoked at resting membrane potential (RMP) by injecting depolarizing current (100 pA for 1 s). **(C)** Enzyme-linked immunosorbent assay (ELISA) analysis reveals elevated level of activin A protein in both wt and dnActRIB hippocampi. A comparison between mice housed in control cages and those experiencing overnight EE revealed strongly enhanced levels of activin A in the hippocampus of the latter (wt control, 0.99 ± 0.03 pg/mg; wt EE, 4.44 ± 0.26 pg/mg; *p* = 2.67e^−15^) (dnActRIB control, 1.15 ± 0.04 pg/mg; dnActRIB EE, 3.52 ± 0.13 pg/mg; *p* = 1.09e^−11^). Numbers in columns indicate sample size. **(D,E)** Plots of action potential (AP) discharge upon depolarizing pulses summarize the EE-induced enhancement in granule cell firing at RMP **(D)** and at a holding potential of −70 mV **(E)**. **(F,G)** EE fails to change granule cell firing in dnActRIB mice. Data from wt controls (in gray) were replicated here to illustrate the unaltered excitability of dnActRIB granule cells. *p < 0.05, **p < 0.01, ***p < 0.001.

When recorded in current-clamp mode, dentate gyrus granule cells (GCs) in brain slices from adult wild type (wt) mice exhibited a strongly hyperpolarized resting membrane potential (RMP; −85.95 ± 0.33 mV, *n* = 86 from 45 mice), which precluded spontaneous firing. In the first set of experiments, we examined the impact of an enriched environment (EE) on the excitability of GCs. While overnight EE-housing had no appreciable effect on RMP (−86.39 ± 0.59 mV, *n* = 18 from 6 mice; *p* = 0.56 vs. control), it increased membrane input resistance (R_N_; at −70 mV: wt-control, 305.31 ± 5.08 MΩ; wt-EE, 335.50 ± 12.78 MΩ; *p* = 0.02). Most importantly, preceding EE experience significantly enhanced AP firing in response to depolarizing current steps of increasing amplitude (50–200 pA, 1 s), applied from RMP or from membrane voltage adjusted to −70 mV (*n* = 18; Figures 1A,D,E).

We next asked whether the EE-evoked rise in activin A and the enhanced firing propensity of GCs are causally linked. To address this question, we took advantage of transgenic mice expressing a dominant-negative mutant of activin receptor IB (dnActRIB), which disrupts activin receptor signaling in a forebrain-specific fashion (Müller et al., 2006). As an important control, we demonstrated that the strong increase in hippocampal activin A after overnight EE was preserved in dnActRIB mice (Figure 1C). For comparison with wt mice, we included here, with additional data points, results from previous ELISA experiments (Dahlmanns et al., 2022). In dnActRIB mice housed in standard cages, the mutant receptor did not produce significant effects on GC firing (*n* = 39 from 15 mice; Figures 1B,F,G), RMP (−85.27 ± 0.57 mV; *p* = 0.71 vs. wt control) as well as R_N_ (−70 mV, 320.69 ± 8.58 MΩ; *p* = 0.12 vs. wt-control). However, EE exposure completely failed to affect GC firing in the mutant preparation (dnActRIB-EE, *n* = 22 from eight mice; Figures 1B,F,G), indicating that intact activin receptor signaling is required to alter GC discharge properties *via* the EE paradigm. Considering the high diffusibility of activin (McDowell et al., 1997), the excitatory effect of this protein on GCs may involve both autocrine and paracrine ways.

### Activin A Excites Granule Cells Through Non-canonical Signaling

**Figure 2:**
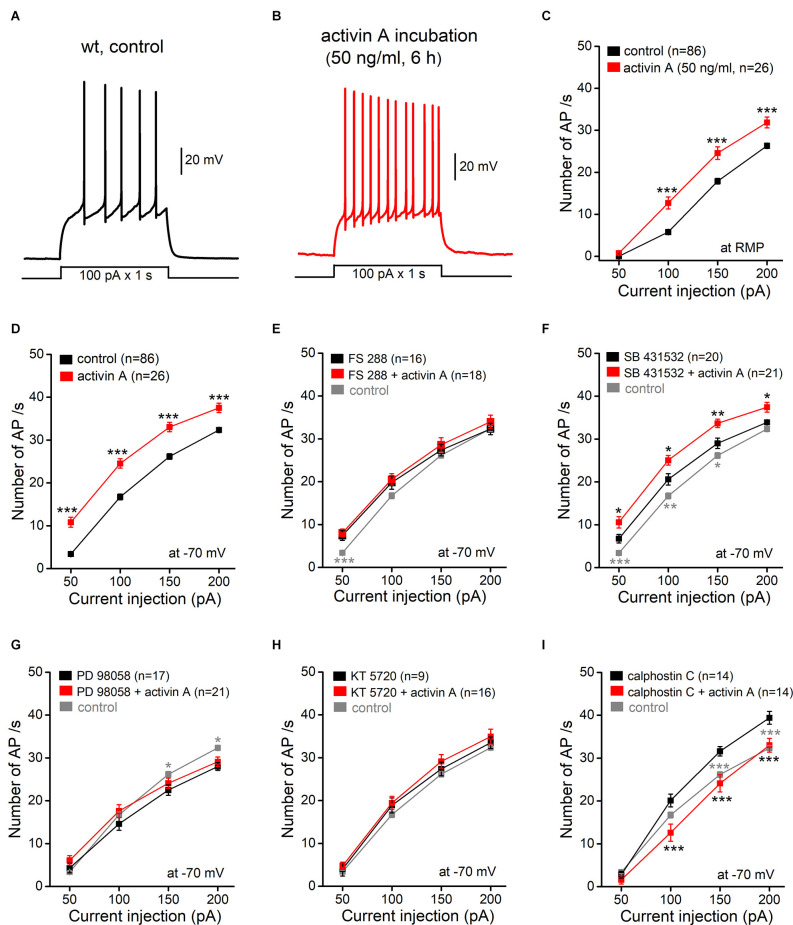
Excitatoryeffect of activin A pre-incubation involvesnon-canonical (non-SMAD) signaling. **(A,B)**Representative current-clamp recordings from a wt control slice**(A)** and from a wt slice pretreated with recombinant activinA (50 ng/ml for 6 h; **B**) at RMP. **(C,D)** Plots summarize theexcitatory effect of activin A (4–10 h) on granule cellfiring upon depolarizing steps (for 1 s) at RMP **(C)** and at −70 mV **(D)**. **(E–I)** Follistatin 288 (FS288, 160 ng/ml; **E**) and SB 431532 (10 μM; **F**)were used to prevent activin receptor binding and to inhibit SMADphosphorylation, respectively. PD 98058 (20 μM; **G**), KT5720 (200 nM; **H**), and calphostin C (100 nM; **I**) wereused to inhibit ERK-MAPK, PKA, and PKC, respectively. For theseexperiments, slices were either incubated with individual inhibitors for several hours or pre-incubated with inhibitors for 30 min before adding activin A (50 ng/ml, 4–10 h). Data from wt controls (in gray) were replicated in **(E–I)** to show the effects of the drugs on cell firing. *p < 0.05, **p < 0.01, ***p < 0.001, compared to the respective black points.

If the rise in endogenous activin during EE renders GCs more excitable, pre-incubation of hippocampal slices from control wt mice with recombinant activin A (50 ng/ml) for extended time periods (4–10 h) should reproduce the excitatory effect of EE. In fact, activin A treatment faithfully mimicked the EE-induced electrophysiological changes in wt granule cells, enhancing AP discharges to depolarizing pulses (*n* = 26 from 11 mice; Figures 2A–D), with a concurrent increase in R_N_ (−70 mV; 344.83 ± 8.96 MΩ; *p* = 0.02 vs. wt-control), but no change in RMP (−85.67 ± 0.62 mV; *p* = 0.54 vs. wt-control). As expected, the effect of recombinant activin A was abrogated, when we repeated the experiment in the presence of follistatin 288 (FS 288; 160 ng/ml; 30 min before activin A), which binds to activin and prevents its interaction with the receptor (Figure 2E; FS 288 alone, *n* = 16 from five mice; FS 288 and activin A, *n* = 18 from four mice). Note that FS 288 alone slightly increased AP firing at 50 pA, which was the lowest current stimulus (7.44 ± 1.13 APs; *p* = 4.6e^−5^, compared to control 3.39 ± 0.40 APs).

In addition to canonical (SMAD-dependent) signaling, activin might also recruit other signaling pathways, including ERK-MAPK, PKA, and PKC signaling. To elucidate the mechanism of activin-induced GC excitation, we used several pharmacological manipulations to dissect the different signaling pathways, beginning with SB 431532, which blocks canonical activin signaling by inhibiting SMAD phosphorylation (Inman et al., 2002). While SB 431532 (10 μM) alone increased AP discharges (at −70 mV: 20.60 ± 1.31 APs at 100 pA, *n* = 20 from five mice; *p* = 0.004 compared to the corresponding control value of 16.73 ± 0.67 APs), the inhibitor did not preclude a further increase in firing rate by activin A, which matched that obtained in control (inhibitor-free) conditions (Figure 2F; SB 431532 and activin, *n* = 21 from four mice). In contrast to the lacking effect of blocking the SMAD pathway, the ERK-MAPK inhibitor PD 98058 (20 μM; Figure 2G; PD 98058 and activin, *n* = 21 from four mice) and the PKA inhibitor KT 5720 (200 nM; Figure 2H; KT 5720 and activin, *n* = 16 from three mice) both abrogated the excitatory effect of activin A on GC firing. Finally, we tested the membrane-permeant specific PKC inhibitor calphostin C (100 nM). Unlike PD 98058 and KT 5720, calphostin C alone made GCs more excitable compared to control (*n* = 14 from five mice), thus complicating the finding that, when examined in its presence, activin A returned the firing-current relationship back to control values (Figure 2I; *n* = 14 from four mice).

**Figure 3:**
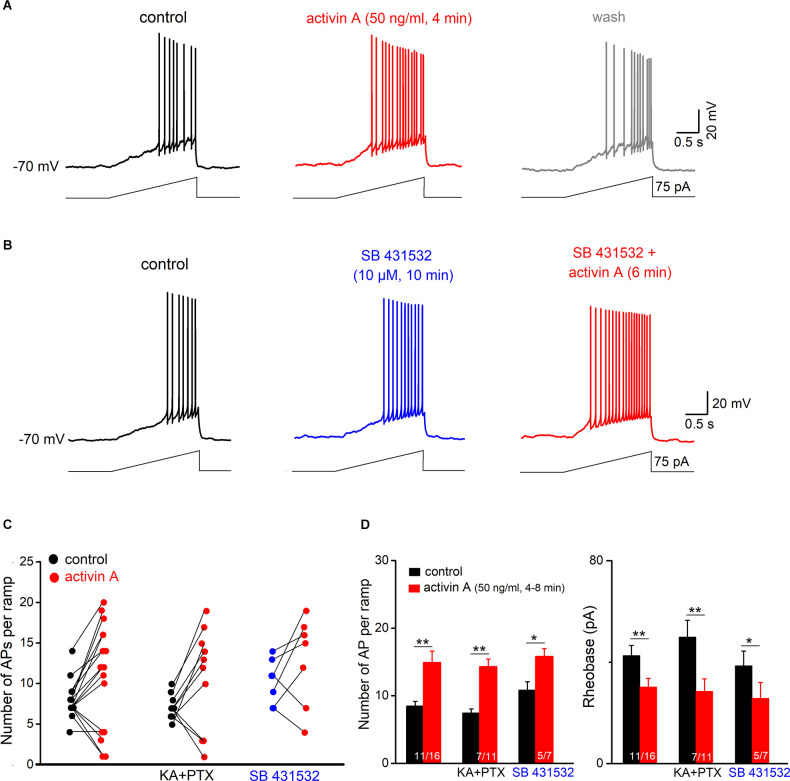
ActivinA excites hippocampal granule cells on a fast time scale. Adepolarizing ramp (rising from 0 to 50–100 pA within 2 s) was usedto test the acute effect of activin A on AP firing in wt granulecells (initially held at −70 mV). **(A)** Voltage traces illustrate the reversible effect of briefly applied activin A (50 ng/ml, 4 min) on granule cell firing. **(B)** Representative traces from a wt slice in which activin A was administered in the presence of SB 431532 (10 μM). **(C)** Summary of granule cell responses to brief application of activin A in control solution (left), in the presence of blockers of GABA_A_ receptors (picrotoxin; PTX) and ionotropic glutamate receptors (kynurenic acid, KA) (middle), and in the presence of SB 431532 (right). **(D)** Histograms summarize enhanced firing and reduced rheobase (the minimal current to evoke first AP) in all granule cells excited by activin A. *p < 0.05, **p < 0.01.

The finding that activin A alters GC firing through a non-canonical pathway which should engender more immediate effects than the classical pathway involving the SMAD transcription factor complex prompted us to explore the action of activin A on a faster time scale. Instead of applying square depolarizing pulses, we now employed a depolarizing ramp protocol to elicit AP firing, which offered the additional advantage to determine rheobase, the minimum depolarizing current to elicit the first AP. For better comparison, membrane potential was pre-set to −70 mV and final ramp current amplitude (50–100 pA) was adjusted to evoke 4–13 APs per ramp (2 s). As shown in Figure 3, superfusion of wt slices with activin A (50 ng/ml) rapidly increased ramp-evoked firing from 8.45 ± 0.72 APs to 14.91 ± 1.71 APs in most GCs (*n* = 11 out of 16, i.e., 68.8%; *p* = 0.002, paired *t*-test; Figures 3A,C,D). Enhanced GC firing was accompanied by a reduction of rheobase (from 42.62 ± 4.02 pA to 30.27 ± 3.34 pA, *p* = 0.002, paired *t*-test; Figure 3D), membrane depolarization (2.74 ± 0.83 mV) and an increase in R_N_ (from 317.64 ± 13.63 MΩ to 365.25 ± 20.64 MΩ; *p* = 0.008, paired *t*-est). In 4 out of 16 tested cells (25.0%), acute activin A decreased firing during ramp test from 6.75 ± 0.25 APs to 2.25 ± 0.75 APs (*n* = 4, paired *t*-est, *p* = 0.006; Figure 3C), concomitant with a reduction in R_N_ (from 256.27 ± 15.43 MΩ to 214.87 ± 11.66 MΩ; *p* = 0.017) and a slight hyperpolarization (−1.44 ± 0.33 mV). The single remaining GC did not change AP firing during activin A application. As almost predicted from the previous experiment, where we had slices pre-incubated with activin A for hours (Figure 2), the excitatory effect of acute activin A was preserved in the presence of SB 431532 (10 μM; five out of seven cells, i.e., 71.4%; Figures 3B–D), which was applied 10 min before activin A to inhibit SMAD phosphorylation. Note that, as with longer incubation, SB 431532 alone increased firing in most GCs (five out eight cells; from 8.20 ± 0.80 APs per ramp to 11.80 ± 0.73 APs, *p* = 0.041, paired *t*-test).

To exclude variable network effects as a source of the apparently divergent actions of acute activin A on GC excitability, we disconnected GCs from their main synaptic inputs by pharmacological means, using antagonists for GABA_A_ receptors (picrotoxin, 100 μM) and ionotropic glutamate receptors (kynurenic acid; 2 mM). In these synaptically silenced slices (*n* = 11; Figures 3C,D), a similar pattern of GC responses to acute activin A was observed, with excitation in the majority of them (*n* = 7, i.e., 63.6%, from 7.42 ± 0.65 APs per ramp to 14.29 ± 1.15 APs, *p* = 0.004, paired *t*-est), whereas the remaining cells were inhibited (36.4%, *n* = 4).

### Activin A Reduces GIRK Current to Excite Granule Cells

**Figure 4:**
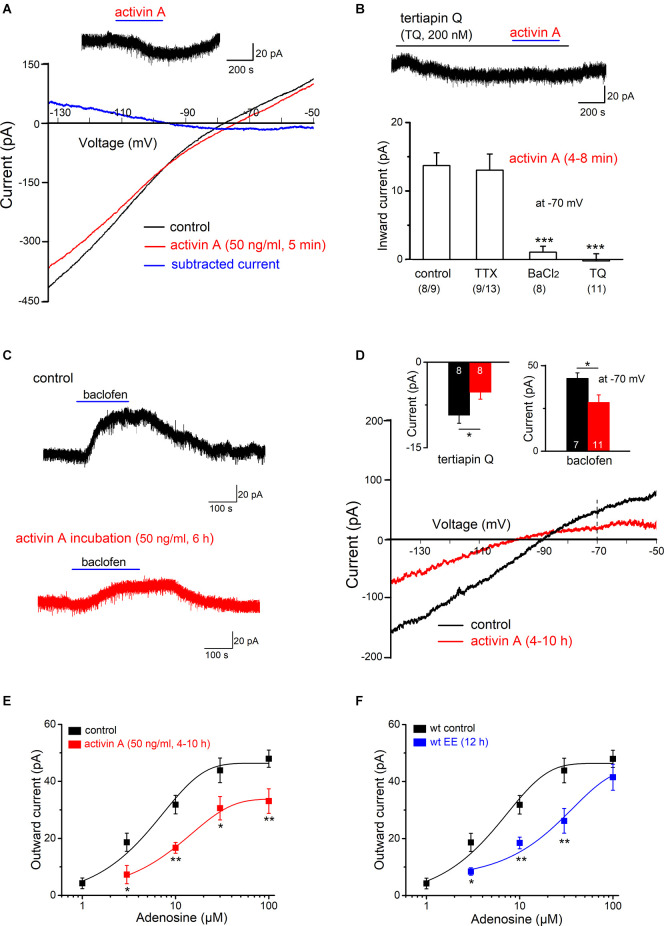
ActivinA reduces GIRK current. Whole-cell voltage-clamp recordings were performed from DG granule cells at Vh of −70 mV. **(A)**Effect of activin A (50 ng/ml for 6 min) on current-voltage (I-V) relationships was determined using a voltage ramp from −50 to −140 mV. Activin-sensitive current (blue trace) was calculated by subtracting I-V curve before (black trace) from that during the maximum effect of activin (red trace). Inset above I-Vcurves depicts the apparent inward current that was reversibly induced by activin A (V_h_ −70 mV). **(B)** Summary of activin-induced inward currents at −70 mV. Activin-evoked inward currents were abrogated by BaCl_2_ (200 μM) or specific GIRK channel inhibitor tertiapin Q (TQ, 200 nM), both applied in TTX (1 μM). Numbers below columns for control and TTX indicate fraction of granule cells displaying an inward current response to activin A. Current trace above the histogram demonstrates that TQ occludes activin suppression of tonic GIRK. **(C)** Sample traces of baclofen (1 μM)-induced outward currents (V_h_ −70 mV) in a control slice (black trace) and a slice pre-incubated with activin A (50 ng/ml, 6 h; red trace). **(D)** I-V curves of baclofen-evoked currents in the absence and presence of activin A pre-treatment. Insets above I-V curves summarize the reduction in baclofen-sensitive outward current (right) and TQ-sensitive inward current (left) in activin A-treated slices (4–10 h). Numbers in columns indicate sample size. **(E,F)** Dose-response curves for adenosine-induced currents in control slices and in slices pre-treated with activin A (4–10 h, **E**) or in slices prepared from EE-exposed mice (12 h; **F**). Data points in **(E,F)** are averages of 5–12 samples. *p < 0.05, **p < 0.01, ***p < 0.001.

To examine the ionic mechanism(s) underlying the effect of activin, we performed voltage-clamp recordings from GCs (V_h_ −70 mV). In the absence or presence of TTX (1 μM) to block AP firing (Figures 4A,B), activin A (50 ng /ml) produced an inward shift of holding current in most granule cells from slices without TTX (13.72 ± 1.87 pA in eight out of nine cells; with no change in the single remaining cell), and with TTX (13.04 ± 1.81 pA in 9 out of 13 cells). In the latter group, few GCs (4 out of 13 cells) exhibited an outward current upon activin A application (17.68 ± 5.77 pA in TTX), consistent with the respective membrane voltage responses in the above current-clamp recordings.

We next used voltage ramps from −50 to −140 mV (in 2 s) to determine the I-V relationship of GCs in the absence and presence of activin A (Figure 4A). Subtraction of the I-V curves demonstrated that activin A (50 ng/ml) suppressed an inwardly rectifying current, which crossed the voltage axis close to the potassium reversal potential (−95.33 ± 4.65 mV, *n* = 8 without TTX; −95.16 ± 6.33 mV, *n* = 9 with TTX). In fact, the K^+^ channel blocker BaCl_2_, which blocks inwardly rectifying K^+^ channels at a concentration of 200 μM, abrogated the effect of activin (*n* = 8, 1.95 ± 0.88 pA at −70 mV; Figure 4B). A potential candidate for the activin-sensitive K^+^ channel is the G protein-gated inwardly rectifying K^+^ channel (GIRK). Since we have previously shown that GIRK current is amplified in CA1 pyramidal cells from dnActRIB mice compared to their wt counterparts (Zheng et al., 2009), it was tempting to speculate that the rise of endogenous activin A (in the EE paradigm) or the application of recombinant activin A (in the slice preparation) produced the opposite effect. To test this hypothesis, we employed the specific GIRK channel blocker tertiapin-Q (TQ, 200 nM) in the presence of TTX. The apparent inward current induced by activin A was indeed almost completely gone in the presence of TQ (*n* = 11, −0.22 ± 1.04 pA at −70 mV; Figure 4B). Since TQ alone evoked an apparent inward current in wt GCs (*n* = 8, 9.30 ± 1.40 pA; inset in Figure 4B) and significantly more so in dnActRIB GCs (*n* = 6, 19.55 ± 2.47 pA; *p* = 0.027), it became obvious that activin A suppresses a standing GIRK current to excite GCs.

### Activin Signaling Dampens Effect of GIRK Current Activators

Activation of GIRK currents is a common mechanism shared by a number of neuromodulators including GABA (*via* GABA_B_ receptors) and adenosine (*via* A1 receptors) to inhibit neuronal excitability (Lüscher and Slesinger, 2010). Thus, we wondered whether long pre-incubation with activin A (50 ng/ml, 4–10 h) would attenuate later GIRK responses to the GABA_B_R agonist baclofen and to adenosine. In control slices in the absence of activin A, baclofen (1 μM) produced an outward current of 42.47 ± 3.34 pA (*n* = 7). This current was significantly stronger than that observed after pre-treatment with activin A (28.59 ± 4.35 pA, *n* = 11; *p* = 0.037; Figures 4C,D). While activin A dampened the GIRK current response to baclofen, it did not affect its reversal potential, which was −92.00 ± 1.60 mV in control cells and −90.70 ± 0.93 mV in cells from activin A-treated slices (Figure 4D). Similarly, adenosine (10 μM) evoked more outward current without activin A (31.83 ± 3.24 pA, *n* = 12) than when the factor was pre-incubated (16.72 ± 1.90 pA, *n* = 6; *p* = 0.006; Figure 4E). Comparison of the dose-response curves to adenosine (1–100 μM) with and without activin A showed that the pre-treatment produced a downward rather than a rightward shift of the curve, leading to a significant decline of the maximal response at 100 μM adenosine (control, 47.96 ± 3.01 pA, *n* = 7; activin A, 30.80 ± 4.34 pA, *n* = 7; *p* = 0.006; Figure 4E). Given that endogenously released adenosine gives rise to a standing GIRK current in the hippocampus (Alzheimer et al., 1993), it was not unexpected that the TQ-sensitive current, which reflects tonic GIRK current, was larger in control cells (9.30 ± 1.40 pA, *n* = 8) than in cells after activin A incubation (5.26 ± 1.21 pA, *n* = 8; *p* = 0.047; Figure 4D, inset).

We next asked whether the significant down-regulation of GIRK current by recombinant activin A in the slice preparation bears significance for the impact of activin in the EE paradigm. Thus, we determined dose-response relationships for adenosine-evoked GIRK currents of GCs in slices prepared from control mice and from mice after overnight EE-housing. As shown in Figure 4F, preceding EE strongly attenuated the GIRK current response to adenosine. This effect strongly resembled that of activin incubation in control slices, with the notable exception that, after EE, the maximum response to adenosine was not reduced, yielding a rightward, rather than a downward shift of the dose-response curve (Figure 4F).

### GIRK Channel Blockade Eliminates Excitatory Effect of Activin on Granule Cell Firing

**Figure 5:**
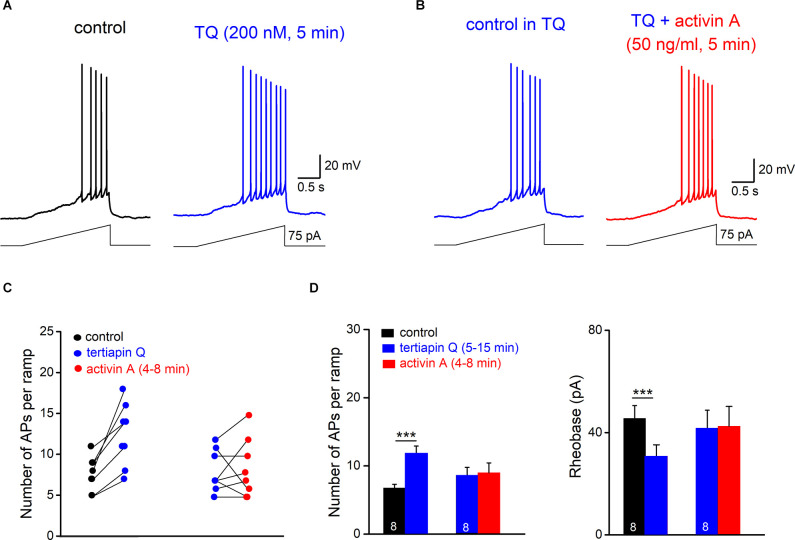
GIRK channel inhibition occludes excitatory effect of activin on granulecell firing. **(A,B)** Tertiapin Q (TQ) alone enhancedramp-evoked firing **(A)** and precluded furtherexcitation by activin A **(B)**. Note that in **(B)** the control trace with TQ (15 min) was reset to −70 mV before activin A was applied. **(C)** Plots of individual granule cell responses to TQ alone and with activin A added later. **(D)** Histograms summarize the abrogation of characteristic effects of activin A on hippocampal granule cell firing and rheobase by TQ. ***p < 0.001.

Based on the above experiments, we predicted that the GIRK inhibitor TQ should both mimic and occlude the effect of activin on granule cell firing. To test this hypothesis, we returned to current-clamp recordings of ramp-evoked firing responses. Like activin A, TQ (200 nM) increased the firing of granule cells during a depolarizing ramp from 6.75 ± 0.56 APs to 11.63 ± 1.28 APs (*n* = 8, *p* = 0.001, paired *t*-est; Figures 5A,C,D), accompanied by a reduction in rheobase (from 45.59 ± 5.09 pA to 30.75 ± 4.48 pA; *p* = 0.001, paired *t*-est; Figure 5D), by an increase in R_N_ (from 314.13 ± 33.33 MΩ to 363.65 ± 35.63 MΩ, *p* = 0.006, paired *t*-est), and by a small membrane depolarization (3.11 ± 0.42 mV). Consistent with the findings obtained in voltage-clamp (Figure 5B), application of activin A (50 ng/ml) in the presence of TQ failed to enhance AP firing in six out of eight cells (75.0%; Figure 5C). Thus, on average, there was neither a significant change in AP numbers per depolarizing ramp (Figure 5D), nor in R_N_ (from 355.13 ± 46.49 MΩ to 353.13 ± 54.82 MΩ; *p* = 0.94, paired *t*-test).

## Discussion

We combined behavioral stimulation of wild type and dnActRIB mice in an overnight EE paradigm with *ex vivo* whole-cell recordings from dentate gyrus GCs to interrogate the role of EE-induced activin A in the regulation of cell firing. Our study shows that EE enhances the excitability of GCs in an activin-dependent fashion. Mechanistically, the increased firing propensity upon membrane depolarization is achieved through non-canonical activin receptor signaling, which led to the suppression of a standing GIRK current. Whereas it is well conceivable that activin signaling employs the canonical (SMAD2/3) pathway to regulate the expression of (unknown) target genes that contribute to the many beneficial effects of EE on cognition and mood, our evidence for a non-genomic mechanism through which activin A inhibits GIRK current is based on the temporal dynamics and the pharmacology of the effect. The increase in GC firing observed after EE exploration was faithfully reproduced by recombinant activin A in hippocampal slices from control animals independent of whether the factor was pre-incubated for hours or acutely applied within minutes. The latter regimen would not be compatible with a signaling pathway involving gene transcription. Moreover, the excitatory effect of activin A was preserved when the factor was pre-incubated with a blocker of SMAD signaling (SB 431542) but was abrogated by a selective ERK1/2 MAPK inhibitor (PD 98058) and a selective PKA inhibitor (KT 5720). A possible involvement of PKC remained ambiguous since the PKC inhibitor calphostin C had a strong excitatory action on its own. That activin induces neurophysiologically significant actions through non-canonical signaling is not without precedent. Hasegawa et al. (2014) showed that acute activin increased the density of large-head spines of hippocampal CA1 pyramidal cells, which facilitated the induction of LTP through weak theta burst stimulation. The rapid effect of activin on dendritic spinogenesis did not require SMAD signaling but was abrogated by inhibitors of ERK/MAPK, PKA, or PKC (Hasegawa et al., 2014).

Since both adenosine- and baclofen-evoked GIRK currents were about equally regulated by activin A, it seems unlikely that the signaling pathway targets the expression or function of adenosine A_1_ or GABA_B_ receptors, respectively. Rather, activin A would be expected to interfere with the molecular processes involved in GIRK channel gating and/or surface expression. It is worth noting that the downward shift of the dose-response curve for adenosine after activin A incubation inversely mirrors our earlier observation with the GABA_B_R agonist baclofen in CA1 pyramidal cells from dnActRIB mice, where genetic disruption of activin receptor signaling produced an upward shift of the dose-response curve for baclofen-induced GIRK current (Zheng et al., 2009). The fact that EE housing caused a rightward shift of the curve for adenosine without altering the maximum response suggests that the EE paradigm induces additional factor(s) that also participate in shaping the GIRK current response to adenosine.

In general, GIRK currents serve multiple neurophysiological functions, and the critical involvement of GIRK channels in many neurological and psychiatric diseases makes them potential therapeutic targets (Kobayashi et al., 2004; Lüscher and Slesinger, 2010; Luján et al., 2014; Jeremic et al., 2021). Owing to their inwardly rectifying current-voltage relationship, constitutive GIRK current or GIRK current tonically activated by ambient concentrations of neuromodulators such as adenosine keep neurons at low RMP and reduce input resistance. Both effects dampen the neuron’s intrinsic excitability (Lüscher and Slesinger, 2010; Kim and Johnston, 2015). Furthermore, dendritic GIRK channels located in close vicinity to glutamatergic synapses are in an exquisite position to affect the flow of excitatory inputs in a fashion that impacts dendritic signal processing and synaptic plasticity (Seeger and Alzheimer, 2001; Takigawa and Alzheimer, 2003; Chen and Johnston, 2005; Malik and Johnston, 2017). Consequently, loss of GIRK current, as reported from GIRK subunit knockout mice, was found to impair multiple brain functions. With respect to the hippocampus, diminished GIRK currents have been linked to learning and memory deficits, reduced anxiety-like behavior, and epileptic seizures (Lüscher and Slesinger, 2010; Victoria et al., 2016; Mett et al., 2021). On the other hand, overactive GIRK channels can also engender cognitive dysfunction, as demonstrated in a mouse model of Down syndrome, in which the mice carry an extra copy of the *Girk2* gene (*Kcnj6*). Hippocampal neurons from GIRK2 trisomy mice display enhanced GIRK current, synaptic plasticity is disrupted, and, at the behavioral level, the mice have deficits in contextual fear learning and recall (Cramer et al., 2010; Cooper et al., 2012). Together, these findings strongly suggest that too little as well as too much of GIRK channel activity is detrimental to hippocampal functions and related mental abilities.

How does the suppression by activin of GIRK current fit into this scenario? It has been argued that the unusually low RMP of GCs, which renders the dentate gyrus a sparsely active network poised for pattern separation, is not only brought about by strong inhibitory circuits, but also by a standing GIRK current (Gonzalez et al., 2018). Does activin then compromise the computational power of the dentate gyrus by inhibiting GC GIRK current? Interestingly, we found that activin A reduced GIRK just to an extent that did not yet affect RMP (where GIRK current is strongest) but made GC more responsive to supra-threshold depolarization, leading to a significantly higher firing rate. In functional terms, this could be understood as an increase in signal-to-noise ratio, which should sharpen the ability of the dentate gyrus to distinguish features of similar input patterns. The reason why a minority of GCs showed an opposite firing response to acutely applied activin A is not immediately obvious. At first glance, it might appear tempting to assume that those cells were immature adult-born GCs, given that they initially lack functional GIRK channels, so that constitutive and neuromodulator evoked-GIRK currents only develop after several weeks of maturation (Gonzalez et al., 2018). This explanation, however, is unlikely, since the fraction of GCs that were inhibited by activin A, did not exhibit the characteristic very high input resistance of new adult-born cells.

Concerns that the enhanced excitability of GC after EE would affect sparse coding in the dentate gyrus might be dispelled by our observation that a rise in activin A produces also an excitatory effect in mossy cells of the hilus (Schmidt et al., 2022). Mossy cells are glutamatergic neurons, which are excited by collaterals of GC axons (mossy fibers). Their axonal projections feed back onto GCs through a direct excitatory pathway and an indirect inhibitory pathway involving the excitation of intermediary GABAergic interneurons. According to recent evidence, the indirect pathway dominates over the direct one in the healthy hippocampus (Botterill et al., 2021). Thus, the concomitant rise in mossy cell excitability by activin A might serve as a safeguard to preserve sparse neural activity as an essential feature of the dentate gyrus, while, at the same time, optimizing the performance of signal processing by GCs.

## Data Availability Statement

The raw data supporting the conclusions of this article will be made available by the authors, without undue reservation.

## Author Contributions

FZ designed and conducted experiments, and performed data analysis. MJV-A and NS performed experiments with adenosine. CA supervised the project. FZ and CA wrote the article. All authors contributed to the article and approved the submitted version.

## Conflict of Interest

The authors declare that the research was conducted in the absence of any commercial or financial relationships that could be construed as a potential conflict of interest.

## Publisher’s Note

All claims expressed in this article are solely those of the authors and do not necessarily represent those of their affiliated organizations, or those of the publisher, the editors and the reviewers. Any product that may be evaluated in this article, or claim that may be made by its manufacturer, is not guaranteed or endorsed by the publisher.

## Abbreviations

aCSF: artificial cerebrospinal fluid
AP: action potential
DG: dentate gyrus
dnActR: dominant-negative activin receptor
ELISA: enzyme-linked immunosorbent assay
ECT: electroconvulsive therapy
EE: enriched environment
FS 288: follistatin 288
GIRK: G protein-gated inwardly rectifying K^+^ channel
GC: granule cell
RMP: resting membrane potential
R_N_: input resistance
TQ: tertiapin Q
TTX: tetrodotoxin.
